# Reinterpreting a community outbreak of *Salmonella enterica *serotype Enteritidis in the light of molecular typing

**DOI:** 10.1186/1471-2458-7-237

**Published:** 2007-09-07

**Authors:** Cristina Romani, PierLuigi Nicoletti, Maria Ida Buonomini, Antonino Nastasi, Caterina Mammina

**Affiliations:** 1 Department of Public Health, University, Via G.B. Morgagni 48, 50134 Florence, Italy; 2 Laboratory of Microbiology and Virology, Azienda Ospedaliero-Universitaria Careggi, Via G.B. Morgagni 85, 50134 Florence, Italy; 3 Centro per gli Enterobatteri Patogeni dell'Italia Meridionale (CEPIM), Department of Hygiene and Microbiology, University, Via del Vespro 133, 90127 Palermo, Italy

## Abstract

**Background:**

In November 2005, a large outbreak due to *Salmonella enterica *serotype Enteritidis (*S*. Enteritidis) was observed within children who had eaten their meals at 53 school cafeterias in Florence and the surrounding area. A total of 154 isolates of *S*. Enteritidis were recovered from human cases between November 2005 and January 2006. All strains were assigned phage type 8 (PT8) and a common *Xba*I pulsotype.

This paper reports the findings of a molecular epidemiological investigation performed on 124 strains of *S*. Enteritidis isolated in the years 2005 and 2006 in Florence and the surrounding area, including the epidemic isolates.

**Methods:**

One hundred twenty-four human isolates of *S*. Enteritidis identified in the period January 2005 – December 2006 were submitted to molecular typing by single enzyme – amplified fragment length polymorphism (SE-AFLP).

**Results:**

Molecular subtyping by SE-AFLP yielded five different profiles. In the pre-epidemic phase, type A included 78.4% of isolates, whereas only three (8.1%) belonged to type C. All isolates, but one, of the epidemic phase were indistinguishable and attributed to type C. In the post-epidemic period, a polymorphic pattern of SE-AFLP types was again recognized but type C accounted for 73.3% of the isolates during the first six months of 2006, whereas during the remaining six months type A regained the first place, including 52.0% of the isolates.

**Conclusion:**

The epidemic event was attributed to the emergence and clonal expansion of a strain of *S*. Enteritidis PT8-SE-AFLP type C. Circulation of the epidemic clone was much more extensive than the surveillance and traditional laboratory data demonstrated.

## Background

Salmonellosis resulting from the ingestion of contaminated food of animal and non animal origin, is a significant public health problem worldwide [1,2]. Foodborne disease outbreaks due to *Salmonella *serotypes are very common in most countries, where this organism has in many cases took advantage from the dramatic changes occurring in the food production chain through the last decades [1,3-6].

*Salmonella enterica *subsp. *enterica *serotypeTyphimurium (*S*. Typhimurium) and *S*. Enteritidis are by far the most prevalent serotypes in humans [2]. *S*. Enteritidis is the most common *Salmonella *serotype globally but especially in Europe, where it accounts for approximately 80–85% of *Salmonella *cases [2]. In particular, Enteritidis has emerged in Italy since the eighties in association with egg-borne outbreaks, consistently with similar findings of a worldwide increase in *S*. Enteritidis infections. Moreover, in many European countries, including Italy, after a decline between the late 1990s and 2002 due to implementation of more stringent European legislation about breeding flocks and improved sanitation and farming practices for egg-laying hens and broilers, the incidence of human infections has again increased starting during the last quarter of 2002 in association with a shift in the prevalent phage types [7,8]. Some countries, like England and Wales and Luxembourg have recently reported a substantial reduction of infection cases due to this serotype [9,10].

Since November 2005, the Azienda Sanitaria Locale of Florence observed a dramatic increase of isolation of *S*. Enteritidis from paediatric cases occurring in Florence and the surrounding area. Public Health Services of Florence, with the technical support of the microbiology laboratory of the "Azienda Universitario-Ospedaliera Careggi" and the Istituto Zooprofilattico Sperimentale of Lazio and Tuscany, started an epidemiological investigation. Children involved in the epidemic proved to be pupils of 53 different schools, who had eaten their meals at multiple school cafeterias contracted out with the same external caterer [11].

The epidemic curve showed a prominent wave consistent with a point common source and a prolonged downslope of the curve including December 2005 and January 2006, that was interpreted as the result of secondary transmission [11].

A total of 154 isolates of *S*. Enteritidis were recovered from human cases between November 2005 and January 2006. Two food handlers tested also positive for *S*. Enteritidis. Attempts to identify food vehicle and trace route of transmission were unsuccessful. All strains were assigned phage type 8 (PT8) and were included into a common *Xba*I pulsotype by pulsed field gel electrophoresis (PFGE) [11].

We report the findings of a molecular epidemiological investigation performed on 124 strains of *S*. Enteritidis isolated in the years 2005 and 2006 in Florence and the surrounding area, including the epidemic isolates. Characterization of the strains has been performed by single enzyme – amplified length polymorphism (SE-AFLP), a molecular subtyping method previously applied on a large selection of sporadic and outbreak isolates of the serotype Enteritidis [12]. The study has been carried out in order to evaluate molecular epidemiological features of the epidemic clone in comparison with the strains recovered from apparently sporadic human cases of infection before and after the epidemic peak.

## Methods

### Bacterial strains

One hundred twenty-four isolates of *S*. Enteritidis identified from human cases of infection in the period January 2005 – December 2006 at the laboratory of Microbiology of the "Azienda Universitario-Ospedaliera Careggi" have been submitted to molecular typing.

All isolates used in this study had been previously identified by the API20E system [Biomerieux, Marcy l'Etoile, France], serotyped by commercially available antisera [Statens Serum Institut, Copenhagen, Denmark] and stored at -70°C.

The strains were classified into three groups: pre-epidemic (January – first week of November 2005), epidemic (second week of November 2005 – January 2006) and post-epidemic (February – December 2006). All available isolates recovered in the pre- and post-epidemic phases, respectively, 37 and 55 isolates, were included in the study. Moreover, 30 isolates were selected by systematic sampling among the 154 isolates from the cases occurring throughout the entire epidemic interval of time and cultured at the "Azienda Universitario-Ospedaliera Careggi". The isolates were stratified by week of isolation and, then, every fifth isolate of the week was included in the study. Both *S*. Enteritidis isolates from the two food handlers were added.

### SE-AFLP

After extraction, DNA was submitted to a simultaneous reaction of digestion with the restriction endonuclease *Hin*dIII and ligation at 37°C for 3–4 h. Adapters H1, 5'-ACGGTATGCCACAG-3' and H2, 5'-AGCTCTGTCGCATACCGTGAG-3' were used. Digested-ligated DNA was heated to 80°C for 10 min to inactivate the ligase, and subsequent PCR was carried out in a 50 μl amplification mixture containing 200 mM each deoxynucleoside triphosphate (dNTP), 2.5 mM MgCl2, 150 ng of each primer and 1 U of *Taq *polymerase.

A mix of four primers was used for polymerase chain reaction (PCR). The primers had the sequence 5'-GGTATGCGACAGAGCTTX-3', where X was either A, T, G or C, respectively. PCR amplification consisted of 1 cycle at 94°C for 4 min, followed by 33 cycles of 94°C for 1 min, 60°C for 1 min, and 72°C for 2.5 min. PCR products were detected on a 1.5% agarose gel run alongside a 100 bp ladder at 100 V for 3 h, followed by staining in ethidium bromide. Photos of the gels were taken under UV light and the gel images were captured in image files. SE-AFLP profiles were analyzed, identified and compared by visual inspection. A difference in at least one band in the range of 600 – 200 bp was adopted as the criterion for type differentiation. Profiles were then classified and labelled using alphabetical letters according to a previously used identification scheme [12].

## Results

**Figure 1:**
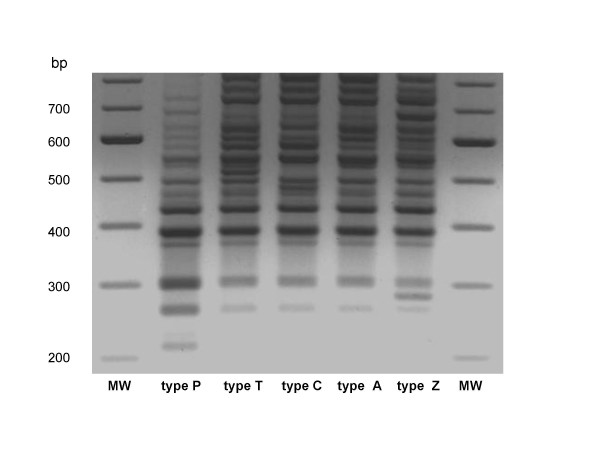
SE-AFLP types detected during the period January 2005 – December 2006.

Molecular subtyping by SE-AFLP of the 124 *S*. Enteritidis isolates under study yielded five different profiles (Fig. 1).

Types A and C were the most prevalent patterns, including, respectively, 37.9% and 51.6% of the strains. SE-AFLP profiles A and C had been previously recognized as the most frequent types SE-AFLP among a large selection of sporadic and epidemic isolates from different regions of Italy [12]. Three new types were also identified and named, respectively, P, T and Z. Only 13 isolates were attributed to these low-prevalence SE-AFLP types.

Analysis of the relative prevalence of the five types in the three periods – pre-epidemic, epidemic and post-epidemic – revealed some relevant differences.

In the pre-epidemic phase, type A included 78.4% of isolates under examination, whereas only three (8.1%) belonged to type C and four (10.8%) and one (1.7%), respectively, to types P and T.

All isolates, but one, of the epidemic phase were indistinguishable and attributed to type C. The single isolate of type A was recovered from a 91 year-aged woman.

**Figure 2:**
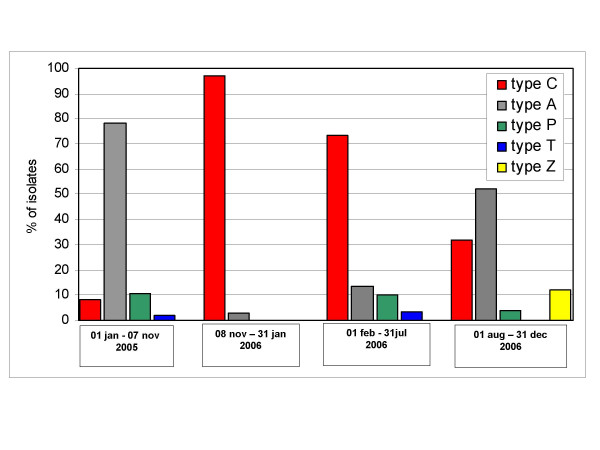
Distribution of SE-AFLP profiles during the pre-epidemic, epidemic and post-epidemic periods.

In the post-epidemic period, a polymorphic pattern of SE-AFLP types was again recognized: type C accounted for 30 (54.5%) isolates, whereas 17 (30.9%) were attributed to type A, four (7.2%) to type P and one (1.8%) to type T. Within type C isolates, eight isolates were retrospectively identified as belonging to a household epidemic cluster occurred in July 2006. A new profile, named Z, was identified in isolates from three members of the same family. Of interest, type C was predominant during the first six months of 2006, accounting for 73.3% of the isolates, whereas during the remaining six months type A regained the first place, including 52.0% of the isolates. However, in this last period, 32.0% of isolates still belonged to type C. Figure 2 illustrates the temporal trend of detection of the SE-AFLP types throughout the interval of time under study.

## Discussion

Since its emergence, *S*. Enteritidis is challenging phenotypic and molecular typing methods, because of the great homogeneity in the genetic make-up of most prevalent strains [12-15]. Time-space defining outbreak events, tracing transmission routes, building microbiologically confirmed causal relationship between human cases and food vehicles have proved to be a very frustrating exercise when dealing with such a serotype [12-15]. Indeed, discriminatory power of the typing techniques has often be unable to reliably differentiate epidemiologically unrelated strains and, eventually, the prominent role of eggs and poultry products as the generally incriminated food items has to some extent withdrawn the interest from a finest strain characterization.

SE-AFLP, a PCR based DNA fingerprinting technique that showed favourable results when applied on a large number of strains of *S*. Enteritidis in terms of discriminatory power, userfriendliness and cost in comparison with phage typing and PFGE, has been applied in this study [12]. Moreover, in the previous study, despite the suboptimal discriminatory power of the technique when applied as a single subtyping approach, a very specific association between SE-AFLP type C and PT2/8 was clearly evident [12].

Some considerations can be drawn from the results of this investigation:

a) the epidemic event was attributable to the emergence and clonal expansion of a strain, that previously was infrequently playing the role of causative agent of enteritis in the geographic area under study. Type C in the pre-epidemic phase had a low prevalence.

b) phage typing, that had been previously applied to all strains isolated during the epidemic peak, included them into PT8. It seems reasonable to conclude that the association PT8-SE-AFLP C characterizes the epidemic clone.

c) the post-epidemic phase appears to be again characterized by a polymorphic pattern, where type C persisted in a prominent position during the first half-year, whereas in the following months type A went back to become again the most frequently identified profile.

d) though in an apparently declining stage, type C was responsible for at least one outbreak in the last months of 2006.

Emergence of type C after a period of low prevalence in the geographic area of interest coincided with an explosive outbreak traced to school cafeterias and, ultimately, to a single caterer. However, beyond the evidence arising from the conventional epidemiological investigations, circulation of the epidemic clone has likely been much more extensive than the surveillance and traditional laboratory data suggested. Indeed, molecular typing support the hypothesis that the epidemic phase did not stop at the end of January 2006. On the contrary, *S*. Enteritidis type C seems to have been protagonist of a hyper-endemic/epidemic exploit until at least the first months of 2006. The slow downslope of the epidemic curve should be reinterpreted based upon the molecular epidemiological evidence: indeed, the previously defined secondary cases appear to arise more likely from a common source that continued over time through the next months.

Moreover, these data strongly support a recent sustained circulation in the geographic area of interest of PT8, historically characterized in Italy as a consistently low prevalence phage type [12].

## Conclusion

Molecular subtyping can significantly contribute to a more reliable interpretation of epidemiological events. Final confirmation of the existence of an outbreak should rely on collaborative interpretation of molecular and conventional epidemiologic data together. Moreover, determining dynamics of disease transmission in widespread areas, distinguishing epidemic from endemic occurrence of transmissible disease and, ultimately, identifying and assessing new and more effective control options could greatly benefit from this synergic approach.

## Competing interests

The author(s) declare that they have no competing interests.

## Authors' contributions

AN, PN and MIB conceived the study. PN and MIB carried out isolation and identification of bacterial isolates. CR performed SE-AFLP typing and analysis of genetic similarity. CM participated in study design, analysis of results and collaborated with AN to draw the conclusions and draft the manuscript. All authors read and approved the final manuscript.

## Pre-publication history

The pre-publication history for this paper can be accessed here:


